# Recent Neurotherapeutic Strategies to Promote Healthy Brain Aging: Are we there yet?

**DOI:** 10.14336/AD.2021.0705

**Published:** 2022-02-01

**Authors:** Chul-Kyu Kim, Perminder S Sachdev, Nady Braidy

**Affiliations:** ^1^Centre for Healthy Brain Ageing, School of Psychiatry, University of New South Wales, Sydney, Australia; ^2^Neuropsychiatric Institute, Euroa Centre, Prince of Wales Hospital, Sydney, Australia

**Keywords:** NAD+, anti-ageing, brain health, caloric restriction, cellular energetics

## Abstract

Owing to the global exponential increase in population ageing, there is an urgent unmet need to develop reliable strategies to slow down and delay the ageing process. Age-related neurodegenerative diseases are among the main causes of morbidity and mortality in our contemporary society and represent a major socio-economic burden. There are several controversial factors that are thought to play a causal role in brain ageing which are continuously being examined in experimental models. Among them are oxidative stress and brain inflammation which are empirical to brain ageing. Although some candidate drugs have been developed which reduce the ageing phenotype, their clinical translation is limited. There are several strategies currently in development to improve brain ageing. These include strategies such as caloric restriction, ketogenic diet, promotion of cellular nicotinamide adenine dinucleotide (NAD^+^) levels, removal of senescent cells, ‘young blood’ transfusions, enhancement of adult neurogenesis, stem cell therapy, vascular risk reduction, and non-pharmacological lifestyle strategies. Several studies have shown that these strategies can not only improve brain ageing by attenuating age-related neurodegenerative disease mechanisms, but also maintain cognitive function in a variety of pre-clinical experimental murine models. However, clinical evidence is limited and many of these strategies are awaiting findings from large-scale clinical trials which are nascent in the current literature. Further studies are needed to determine their long-term efficacy and lack of adverse effects in various tissues and organs to gain a greater understanding of their potential beneficial effects on brain ageing and health span in humans.

## 1.Introduction

### 1.1.*Why Population Ageing Matters*

In the past century, the life expectancy of humans has almost doubled in developed countries due to improved healthcare, nutrition, and effective antibiotics against infectious diseases. In the United States alone, it has been estimated that today’s 65-year-olds can live for a further 19.4 years, or 5.5 years longer that 65-year-olds in the 1950s. The number of people over 65 years of age in the United States is expected to reach 98 million by 2060 (currently 46 million), or 25% of the total population. Age-related disorders such as cardiovascular disease, cancer, and neurodegenerative diseases are the primary causes of morbidity and mortality both nationally and abroad [1-7]. Unfortunately, the outcomes of brain health are not harmonised with the outcomes of lifespan extension.

Progressive ageing of tissue, cells and organs is associated with a gradual decline in function during the lifespan of an organism. Numerous studies have shown that physical frailty is associated with low cognitive function and mild cognitive impairment (MCI) [8-11]. MCI is a term used to describe the stage between the expected cognitive decline of normal ageing and the more serious pathological decline leading to the dementias, and includes impairments in learning, memory, language, thinking and judgment that exceed normal age-related changes. The severity of physical frailty is likely to predict a worse cognitive trajectory among participants with MCI and it is linked to a greater risk of developing MCI [12].

The process of developing and maintaining the functional ability that enables wellbeing in older age is defined as “healthy ageing”. More specifically, older individuals in their sixties, seventies, and eighties that age well do not show significant decline in physical and cognitive performance and are active in their lifestyle. Lifespan extension is the primary goal of anti-ageing research. However, a greater importance has been placed on maintaining physical and mental health during ageing since ageing is a major risk factor for age-related degeneration and neurocognitive disorders, which not only affect the quality of life of individuals but also their family members and carers and the global economy [13]. Identifying and developing strategies aimed at preventing the occurrence of age-related neurodegenerative diseases is crucial. Therefore, development of interventions that slow down the rate of ageing and reduce or postpone the incidence of debilitating age-related neurocognitive disorders are of considerable value to improve the quality of life and reduce medical costs [14, 15]. Studies in animal models have identified a variety of molecular mechanisms that are likely to lead to interventions which enhance lifespan and reduce cognitive decline [16-18]. In this review, we summarise mechanisms and effectiveness of recent anti-ageing strategies, using findings from recent animals and human studies, and highlight how they may contribute to brain health. We also examine how these strategies may represent a promising therapeutic strategy to counter ageing-associated pathologies in the brain and slow down and/or attenuate age-related cognitive decline.

### 1.2. Molecular mechanisms of brain ageing, biomarkers and potential intervention

Ageing has a profound negative impact on the brain and cognitive performance[19]. Ageing can affect cortical neurotransmission and synaptic function, neurogenesis, vasculature, gross morphology, and cognition via multiple processes. It is well established that as we age, the brain recedes in volume, particularly in the frontal cortex. Our aging vasculature can lead to elevated blood pressure and increased risk of stroke and ischemia and white matter lesions. Memory deficits also occur with advanced aging and brain activation becomes more bilateral for memory tasks, to compensate and recruit additional networks. Genetics, neurotransmitters, hormones, and experience all play a role in brain aging. However, higher levels of education or occupational attainment may slow down brain aging. As well, leading a healthy lifestyle including consuming good nutrition, low to moderate alcohol intake, and regular exercise exert a protective effect against brain aging.

**Figure 1.**
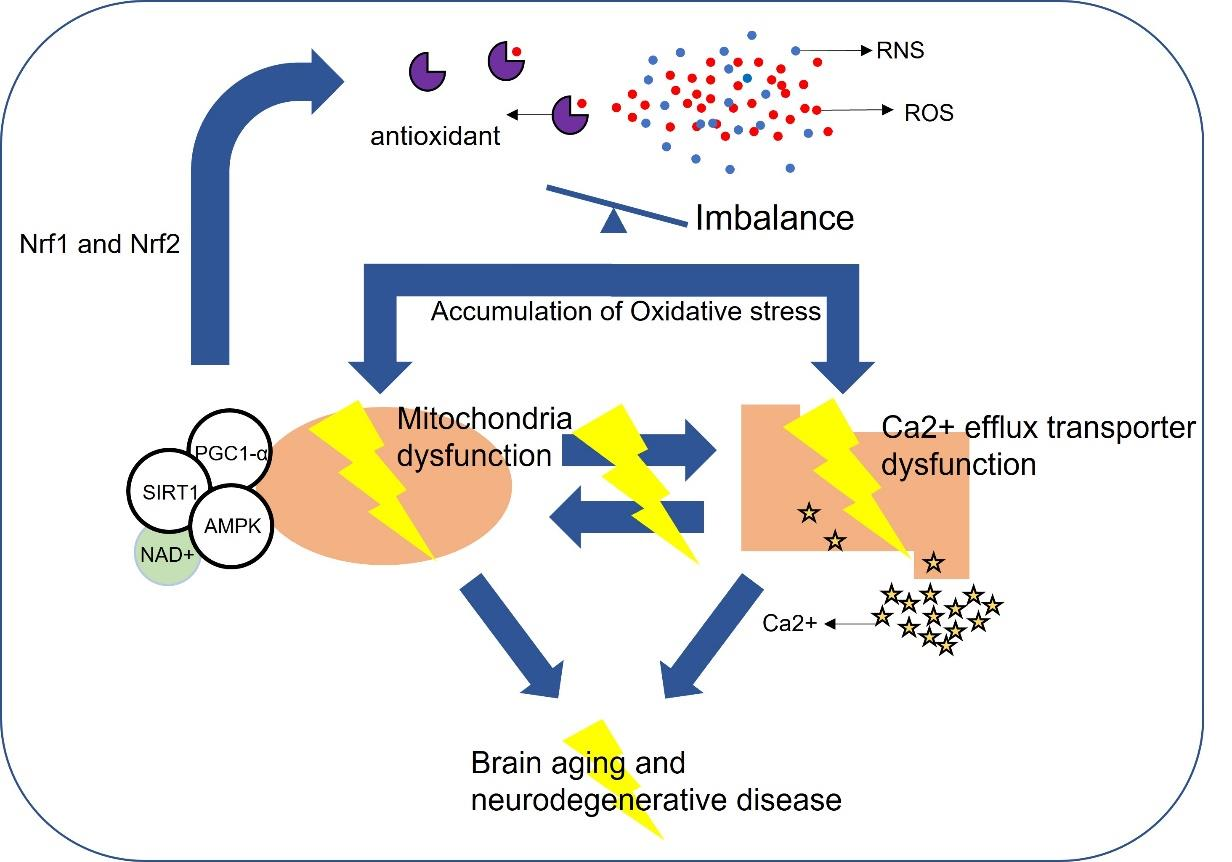
Oxidative stress induced by an imbalance in ROS production can accelerate brain aging. Overload of RNS and ROS is the main factor leading oxidative stress. PGC-1α which is activated by AMPK and SIRT1 interacts with Nrf1 and Nrf2. Nrf2 plays a critical role to regulate antioxidant activity in the mitochondria. Imbalance between ROS and antioxidant can cause oxidative stress. This imbalance causes mitochondrial dysfunction and Ca2^+^ efflux transporter deficits. Mitochondria dysfunction is an important factor of brain aging and can impair Ca2^+^ efflux transporters. Ca2^+^ efflux transporter dysfunction promotes permeability of mitochondria and activates proapoptotic pathways. This mechanism can cause negative effects on brain aging.

Several factors that contribute to age-related decline in the brain have been previously discussed [20-23]. Oxidative stress is a critical factor in the aging brain (Fig. 1). The brain is especially vulnerable to oxidative stress compared to other organs. This is because it has a high-energy demand and processes approximately 20% of basal O_2_ consumption in humans [24]. Oxidative damage to tissues, cells and organs occurs when there is an imbalance in the generation of reactive oxygen species (ROS) and reactive nitrogen species (RNS), and the bodies endogenous antioxidant defence mechanisms. The ROS and RNS balance and redox regulation are integral to maintaining normal brain homeostasis. ROS/RNS can affect not only the immune response and inflammation, but also synaptic plasticity, learning, and memory [25]. Furthermore, the accumulation of oxidative stress can trigger damage to lipid, protein and nucleic acids [26, 27]. For instance, nuclear DNA (nDNA) and mitochondrial DNA (mtDNA) oxidation, modification of proteins, lipid peroxidation of membranes, and mitochondria dysfunction are induced by oxidative stress leading to accelerated brain aging, neuronal loss and cognitive impairment [28]. ROS can affect factors related to the pathobiology of neurodegenerative diseases such as hyperphosphorylation of tau and misfolding of amyloid beta (which are key components of intracellular neurofibrillary tangles (NFTs) and extracellular amyloid plaques), alpha-synuclein (present in Lewy bodies), and mutant Huntington protein. There is a strong association between these misfolded protein and neurodegenerative entities in Alzheimer’s disease (AD), Parkinson disease (PD), and Huntington disease (HD), respectively [29-31]. Mitochondrial dysfunction induced by oxidative stress can greatly contribute to physical and cognitive changes in the brain. Mitochondria are particularly sensitive to oxidative stress because they generate large amounts of ROS [32]. Mitochondrial dysfunction is specifically critical in organs where demand for energy is high [33]. Neuronal mitochondria play a crucial role in the brain such as regulating stress reactions and maintaining metabolic homeostasis [34]. Since the mitochondria is an important organelle, mitochondrial dysfunction can affect the brain significantly [35, 36]. For instance, mitochondrial dysfunction can increase the risk of AD via accumulation of amyloid beta [29, 37], and the risk of PD is associated with dysfunction of mtDNA and the mitochondria [33].

Peroxisome proliferator-activated receptor gamma coactivator-1α (PGC-1α), which is activated by sirtuin-1 (SIRT1), is a key regulator of mitochondrial biogenesis. PGC-1α is associated with nuclear factor erythroid 2-related factor 1 (Nrf1) and nuclear factor erythroid 2-related factor 2 (Nrf2), responsible for ROS detoxification [38]. The transcription factor Nrf2 modulates the level of antioxidant defences in mitochondria as well as is a key regulator of inflammation. Additionally, SIRT1, an NAD+ dependent deacetylase, is triggered by Adenosine Monophosphate-activated Protein Kinase (AMPK) through increasing NAD+ levels [39]. Sirtuins are known to be involved in processes such as DNA repair, neurogenesis, inflammation, metabolism, mitochondria homeostasis, autophagy, apoptosis, oxidative/anti-oxidative balance, and aging [40]. Among them, SIRT1 regulate forkhead box O (FOXO), p53, PGC-1α, and nuclear factor-κB (NF-κB) [41]. The induction of NAD+/SIRT1 and autophagy regulation by AMPK inhibited cellular senescence [42]. For example, AMPK activity not only prevented H_2_O_2_-induced senescence but also improved the impaired autophagic flux via promotion of NAD+ synthesis [43]. In *Caenorhabditis elegans*, AMPK improved the dysfunction of mitochondrial networks induced by age [44].

The accumulation of ROS also causes calcium ion (Ca^2+^) overload throughout the body and damages Ca^2+^ efflux transporters [45]. Moreover, since mitochondria and endoplasmic reticulum (ER) play a crucial role in the regulation of Ca^2+^, mitochondrial dysfunction also affects the imbalance of Ca^2+^ homeostasis [46, 47]. Although increasing Ca^2+^ level is associated with ATP generation, the overload of Ca^2+^ can stimulate apoptotic pathways and increase the permeability of the mitochondrial membrane [48]. Thus, excessive Ca^2+^ is shown to have a cobweb-like association with increasing mitochondrial damage and generation of ROS [49]. The imbalance of Ca^2+^ homeostasis can trigger age-related loss to neuronal performance and other molecular pathways leading to aging and death [50]. Ca^2+^ imbalance can also play a causal role on cognitive function and lead to a variety of pathologies [51, 52]. Additionally, Ca^2+^ homeostasis is related to age-related cognitive deficits as well as neurodegenerative diseases [24, 26].

Additionally, the Insulin/insulin-like growth factor 1 (IGF-1) signalling pathway has been identified as another factor associated with aging. Insulin produced throughout the liver induces IGF-1. Produced Insulin and IGF-1 can be transported by lipoprotein receptor-related protein-2 (LRP2) and can cross the blood brain barrier to enter the brain. Insulin and IGF-1 can bind to the IGF-receptor and insulin-receptor, which is phosphorylated to be activated. Moreover, IGF-receptor and insulin receptor can combine and then bind to both insulin and IGF-1. This phenomenon affects the cell stress response and metabolism related to phosphoinositide 3-kinase (PI3K)/protein kinase B (AKT), mammalian target of rapamycin (mTOR), mitogen-activated protein kinase (MAPK)/extracellular signal-regulated kinase (ERK), and FOXO signalling [53-55]. Decreased IGF-1 in the brain is highly associated with brain aging. IGF-1 is normally known as a positive factor in the brain. High levels of IGF-1 may be neuroprotective and maintain cognitive function [56, 57]. Short-term exposure of IGF-1 in mice showed recovery of learning and memory [58]. In a murine AD model, increasing insulin and IGF-1 reduced the accumulation of amyloid beta, which is connected to MAPK signalling [56].

Inflammation in the brain can be increased with age and disease [59]. The genetic and environmental factors of inflammation have been shown to accelerate aging and age-related diseases [60, 61]. For example, neuroinflammation is associated with the pathobiology of AD. Peripheral inflammation has been associated with cognitive decline and dementia at a certain age. Meanwhile, high inflammatory levels correlated to higher mortality in the elderly [62, 63]. Key inflammatory players in aging brain include activated cytokines, immune cells, microglia, astrocytes, brain-derived neurotrophic factor, and IGF-1 transport [64]. Among them, microglia are resident immune cells and key regulators of neuronal and synaptic function including protection and vascular re-modelling in the brain [65]. In addition, microglia is especially associated with regulating the levels of pro-inflammatory cytokines including interleukin 1β (IL-1 β), interleukin 6 (IL-6), and tumor necrosis factor alpha (TNF-α) [66]. Moreover, the neurotrophic factors derived by microglia are crucial for cognition. Microglial depletion decreased neuronal loss in AD mouse model [62].

Biological aging is unlikely to be tied absolutely with chronological aging. Recent strategies have been developed to potentially slow biological aging and lower the possibility of suffering from age related neurodegenerative diseases including the dementias. Several anti-aging strategies that can promote healthy brain aging are in development. This review examines the efficacy of the emerging anti-aging approaches for maintaining better brain function. These approaches include strategies such as caloric restriction, ketogenic diet, promotion of cellular nicotinamide adenine dinucleotide (NAD+) levels, removal of senescent cells, ‘young blood’ transfusions, enhancement of adult neurogenesis, stem cell therapy, vascular risk reduction, and non-pharmacological strategies, such as physical activity.

## 2.Method

*2.1 Search strategy*

This systematic review followed the guidelines of Preferred Reporting Items for Systematic Review and MetaAnalyses (PRISMA). A systematic electronic search was conducted using PubMed Medline, Web of Science, and Embase (dated January 2018 to July 2021). The search was restricted to research articles that examined recent strategies for brain aging including CR, ketogenic diets (KD), nicotinamide riboside (NR), senolytics, ‘young blood’ transfusions, adult neurogenesis, stem cell therapy, vascular risk reduction, and non-pharmacological strategies. The search used the following keywords: CR, KD, NAD+, nicotinamide riboside, senolytics, blood transfusion, parabiosis, neurogenesis, vascular risk, hypertension, non-pharmacological, cognitive stimulation, brain health, brain aging, cognitive training, working memory, executive function, cognitive enhancement, elderly, and healthy older adults.

### 2.2 *Inclusion/exclusion criteria*

We only considered research articles which reported the impact of anti-aging strategies (CR, KD, NAD+, senolytics, ‘young blood’ transfusion, adult neurogenesis, stem cell therapy, vascular risk, hypertension, non-pharmacological, cognitive stimulation) on the brain or neurodegenerative diseases, and those published from 2018 to 2021 were included in order to provide up-to-date review. Review articles were excluded. Research articles that examined the impact of the above strategies on the brain but were not related to aging or neurodegenerative diseases were also excluded.

### 2.3 Data extraction and data items

The animal species/type of animal model or human clinical trial, sex, number of subjects, and functional outcomes on the brain physiology and cognition were included as data. Additional data were extracted to suit each strategy such as diet intervention for CR and KD, treatment and dose for senolytics, and adult neurogenesis, used serum and treatment for ‘young blood’ transfusion, cell type used in stem cell therapy, vascular risk factors, and non-pharmacological strategies.

## 3.Results and discussion

### 3.1 Study selection

**Figure 2.**
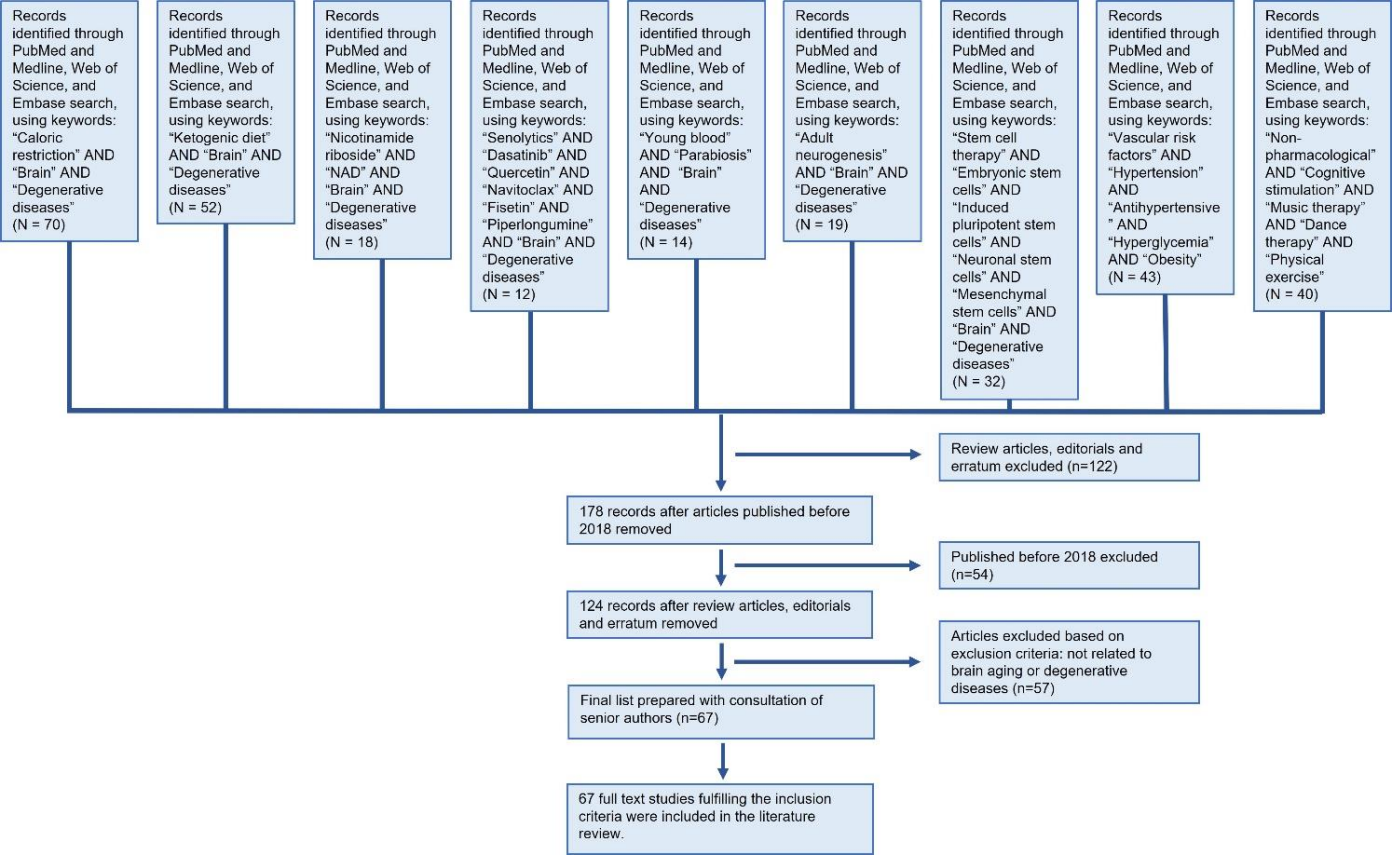
The selection process of inclusion or exclusion of articles.

Figure 2 summarises the search strategy. A total of 300 studies were identified after searching with keywords. Subsequently, 122 review articles including editorials and erratums, and a further 54 studies published before 2018 were excluded. After that, 124 studies were remained. Since 57 studies did not meet our selection criteria, they were excluded. A total of 67 studies were included in this review.

### 3.2 Study characteristics

All the studies included in the review described the impact of anti-aging strategies on the brain or neurodegeneration. This review describes what the anti-aging strategies are and how they have been shown to impact on brain health and cognition.

### 3.3 Recent strategies to improve brain aging

#### 3.3.1 Caloric restriction

CR has been defined as a strategy to reduce calorie intake by 10 - 40% without malnutrition. CR has been shown to extend the life span and health span in diverse animal species from yeast to primates [93-98]. This dietary intervention showed effects including improving general health, preventing various diseases, and attenuating cognitive deficits in memory and learning. Several age-related diseases including chronic inflammatory disorders and neurological diseases were also protected by CR in animal models [99, 100]. Furthermore, CR might extend health span to prevent various pathological conditions including cardiovascular disease, diabetes and cancer by retarding the onset of these diseases [101, 102]. The intervention has been enthusiastically studied (Table 1).

**Figure 3.**
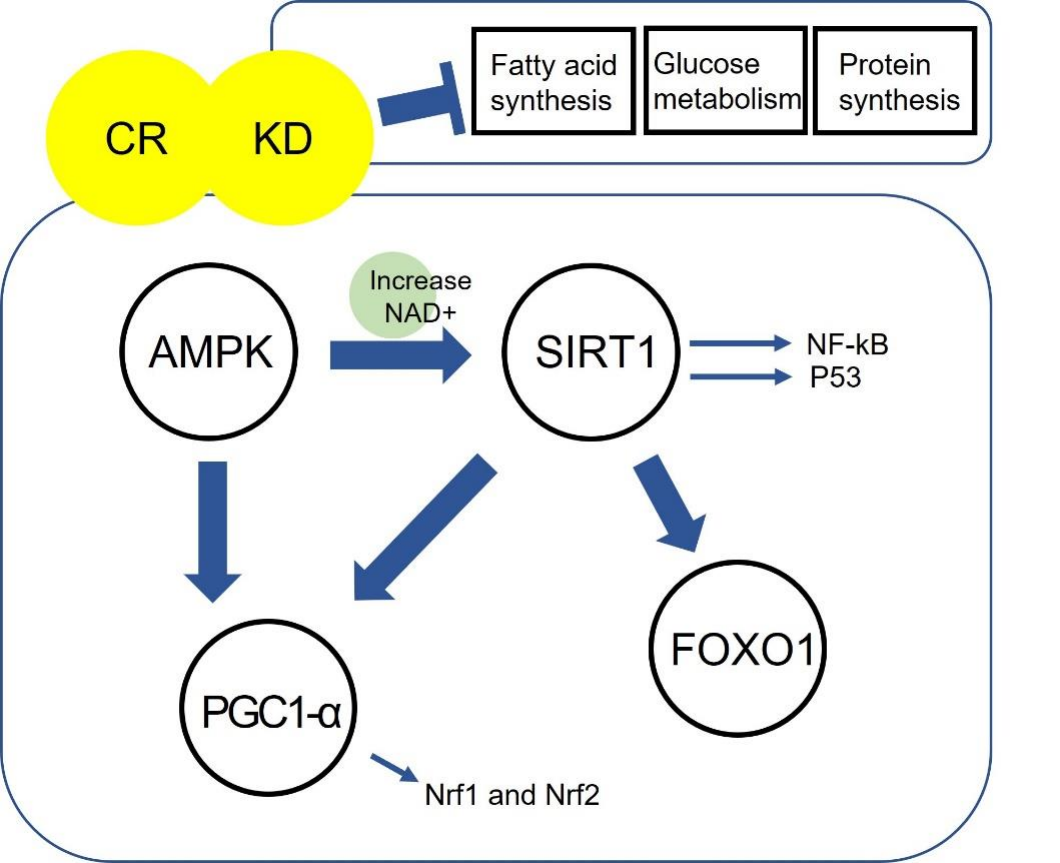
Proposed mechanism of intervention of CR and KD in brain ageing. CR is associated with AMPK, SIRT1, PGC-1α, and FOXO1 pathways. These signalling pathways are inter-related. SIRT1 and increased NAD+ is regulated by AMPK. SIRT1 can activate PCG1 α which regulates Nrf1 and Nrf2. Nrf1 and Nrf2 work as antioxidants. SIRT1 is also associated with NF-κB, p53, and FOXO1. These relationships are essential factors for anti-brain ageing and protective activity in neurodegenerative disease. KD has similar mechanisms as CR. KD can also inhibit Fatty acid synthesis, glucose metabolism and protein synthesis. These factors may be associated with the pathobiology of AD, PD and HD.

The mechanism of CR is still unclear and remains controversial. However, there are a lot of hypotheses to explain the effects of CR including autophagy, apoptosis, mitochondrial activity, redox homeostasis, mTOR signalling, AMPK, and Sirtuin [103-106]. When calories are restricted, more carbons are oxidized in mitochondria via the electron transport chain-mediated cellular respiration, which produces NAD from NADH [107, 108]. Thus, under caloric restriction, the NADH levels are significantly decreased as a result of up-regulated mitochondrial respiration [109, 110]. Recent studies support the hypothesis that CR is associated with several aging pathway such as PGC-1α, SIRT1, and AMPK pathway which are dependent on the essential pyridine nucleotide, NAD+ (Fig. 3). SIRT1, one of key target factors in CR, is an NAD-dependent histone deacetylase that has multiple roles including life span extension, stress resistance, and reduction of apoptosis [111].

**Table 1:** Recent studies on caloric restriction and brain health.

Animal		Number of animals	Diet	Functional outcome	Ref*
Animal type	Sex				
Mouse	Male	4-5 mice per group	30% less calories	- increased SIRT1 mRNA level in the hippocampus - increased FOXO1 mRNA level in the hippocampus	[119]
	Male	12 mice per group	30% less calories	- improved learning and memory - increased the level of IGF-1 protein - decreased glucose and malondialdehyde level in the serum - increased the number of AMPK and GLUT4 and the mRNA of those in the brain	[120]
	Male	8 mice per group	10% less calories at 14 weeks 25% less calories at 15 weeks 40% less calories at 16 weeks	- decreased the age-related CG methylation in the hippocampus - decreased the age-related CH methylation in the hippocampus	[407]
	Both	12 mice per group	20% less calories	- increased SIRT1 protein expression in female mice - increased PGC-1a protein expression in male mice - improved recognition indices of female mice in the novel object recognition test	[121]
	Male	5-12 mice per group	30% less calories	- improved sensorimotor function following ischemic injury - improved cognition and memory after ischemic injury - protected white matter tracts and neuron following ischemic injury	[124]
	Male	14 mice per group	40% less calories	- increased neurotransmitters - increased neuronal integrity markers - increased essential fatty acids - increased biochemicals associated with carnitine metabolism	[116]
	Male	3-11 mice per group	10% less calories	- prevented the cognitive impairment in traumatic brain injury mice model - increased SIRT1 protein levels in the cortex and the hippocampus in traumatic brain injury mice model	[122]
	Male	20 mice per group	40% less calorie for 12 weeks	- improved memory - increased SIRT1 and HSP70 mRNA expression in the hippocampus	[118]
	Both	5-10 mice per group	10% less calories at 14 weeks 25% less calories at 15 weeks 40% less calories at 16 weeks	- increased subventricular zone stem cell proliferation in young mice - prevented the loss of neurogenesis in aged mice - improved olfactory memory - decreased microglia expression - decreased the level of inflammation marker in the subventricular zone	[123]
Rat	Male	5 rats per group	40% reduction in food intake	- decreased glucose levels in the serum - increased AMPK and pAMPK levels in the cortex and hippocampus of the aged rats - decreased cholesterol precursors, lathosterol and lanosterol, in both hippocampus and cortex of the aged rats	[125]
	Male	45 rats	30% less calories	- improved acrolein-induced cognitive impairment - protected acrolein-induced GSH deletion in both cortex and hippocampus - improved acrolein-induced SOD activity decline in the hippocampus - positively regulated AD-associated proteins	[128]
	Male	7-8 rats per group	30%-40% less calories	- decreased total oxidant status in the brainstem, cerebellum, frontal lobe, parietal lobe, and hippocampus. - improved antioxidative capacity (Cu, Zn-SOD) in the frontal lobe - decreased the rate of lipid hydroperoxides formation in brain tissue	[126]
	Male	19 rats per group	40 % less calories daily for 11 months	- alleviated decrease the thiol level in the hippocampus, parietal cortex, and cerebellum - increased GSH concentrations in the hippocampus, striatum, and cerebellum - increased GSH peroxidase activity in the hippocampus and parietal cortex - increased GSH reductase activity in the hippocampus	[117]
	Male	11-19 rats per group	30% reduction in food intake	- improved the long-term memory of aged rats	[127]
Human		Number of subjects treated	Diet	Functional outcome	Ref*
Trial type	Sex				
Randomized order	Female	17	Very low-calorie diet (511 kcal/day)	- increased accuracy in the MSTT (Matching to sample test Reaction time) - decreased accuracy in the CRTT (Choice reaction time test Reaction time)	[130]
Parallel group, randomized clinical trial	Both	Total 220	25% reduction of the subject's regular calorie intake for 2 years	- improved performance in SWMS† - improved SWMTE at month 24 - improved working memory (measured by SWMTE‡)	[129]

* These references were published from 2018.

† (SWMTE: This is the number of times a box is selected that is certain not to contain a blue token and therefore should not have been visited by the subject, ie, between errors þ within errors - double errors)

‡ (SWMS: For problems with 6 boxes or more, the number of distinct boxes used by the subject to begin a new search for a token, within the same problem)

CR showed impact on neurodegenerative diseases by reducing the number and size of amyloid beta plaques in AD transgenic animal models [104, 112]. In mice, cognitive function and long-term memory were improved by CR. As well, CR slowed the age-related mitochondrial function and maintained neuronal activity [113, 114]. In addition, CR decreased the accumulation of amyloid beta in an AD mouse model [104]. The intervention is shown to improve mitochondrial activity in rat cells by reducing ROS production, and this is associated with cognition [115]. Consequently, CR prevented decline of memory and cognition as well as the onset of neurodegenerative diseases in rodents [116, 117].

In mice, CR increased the mRNA level of SIRT1 and FOXO1in the hippocampus [118, 119], and the mRNA level of AMPK in the brain[120]. SIRT1 protein expression in the female and in traumatic brain injury model were increased by CR [121, 122]. CR showed increase of PGC-1α protein expression in male (Wahl et al., 2018) and IGF-1 protein level in serum [120], whereas CR decreased microglia expression and inflammatory markers [123]. Furthermore, the intervention also showed various beneficial effects such as enhancing recognition indices and olfactory memory [121, 123] and improving cognition, memory, and sensorimotor function after ischemic injury [124]. In rats, CR increased AMPK and pAMPK [125], glutathione (GSH) concentration, GSH peroxidase activity, and GSH reductase activity in specific parts of the brain in aged rats [117]. Additionally, the total oxidant status was observed to be at a lower rate in CR model while the antioxidative capacity such as Cu, Zn-SOD was improved [126]. Moreover, cognitive impairment, GSH deletion, and impaired SOD activity induced by acrolein were positively regulated and long-term memory of old rats was improved by CR [127, 128].

Most CR studies in animal models used male mice. However, one study using both sex mice identified sex-dependent effects of CR on brain aging [121]. For example, CR increased SIRT1 expression only in female mice, while PGC-1α expression increased only in male mice [121]. Sex is a limiting factor in CR studies in humans as well. For example, one study reported findings in women only, while another group studied both men and women, but the study is also limited because women distribution of sample was predominant [129, 130].

In humans, CR was shown to induce body weight loss, reduce mortality and improve general health, sleep quality and sexual function [129, 131, 132]. This enthusiastically studied intervention also displayed improved cognition in humans [129, 130]. The brain aging and degenerative diseases might be prevented by CR, while the brain factors of the age-related decline including long-term potentiation (LTP) and brain-derived neurotrophic factor (BDNF) were reduced [129, 133, 134]. However, evidence from human interventional studies is limited. [133] reported improvements in verbal recognition memory performance in healthy older normal to overweight subjects who were instructed to reduce calorie intake by 30% over a 3-month period. Memory improvement was associated with improved glucose metabolism and lower fasting plasma insulin concentration [135]. Most human studies involving CR report most effects improved energy homeostasis. Therefore, CR is likely to improve brain health by mimicking the effects of short-term negative energy balance [136], rather than reduced weight. However, it remains unclear whether the benefits of CR remain stable over time or are linked with negative energy balance during the weight loss phase. The issue of sustainability is of considerable importance as chronic CR has reported limited adherence [137], has not always demonstrated benefits on cognitive function (e.g., [138, 139]. and at times may present negative health effects in subjects with incipient dementia [140].

A recent two-year randomized controlled trial study reported that CR shows no significant side effects on factors related to quality of life including mood, self-reported hunger, sexual function, and cognition [141]. Moreover, mild CR for 2 years also showed no side effects on assessments of vitality, mental health and bodily pain (SF-36) [131]. These studies can support the safety of the intervention. However, it is still unclear how much caloric intake is ideal for optimal health.

#### 3.3.2 Ketogenic diet

KD is a recent dietary intervention that is very high in fat and low in carbohydrates. The intervention was firstly initiated to reduce the symptoms of epilepsy. In rodents, KD not only improved memory in mice and cognition in rats but also reduced amyloid beta levels and cell death [142-144]. Moreover, KD showed improvements in overall brain function and stability in humans [145]. The KD has been reported to promote positive effects on brain aging and neurodegenerative diseases such as AD, PD, and HD [146, 147]. Recent studies showed positive effects of KD not only in animal models but also in humans (Table 2). KD demonstrated effects including decreasing mTOR protein expression and encouraging amyloid beta clearance in mice [148] as well as behavioural and cognitive enhancement [144] and increasing anti-aging factors such as the NAD+/NADH ratio and intracellular NAD+ level and NAD-dependent processes including sirtuin activity, and SIRT1 gene expression in rats [149]. In humans, memory and cognition were also improved by KD [150-152], in the patients with diabetes [153], with HIV [154], and with mild AD [155].

**Table 2:** Recent studies regarding ketogenic diet on brain health.

Animal		Number of animals	Diet	Functional outcome	Ref*
Animal type	Sex				
Mouse	Male	9-10 mice per group	75.1% fat, 8.6% protein, 4.8% fiber, 3.2% carbohydrates, 3.0% ash,	- decreased mTOR protein expression - improved neurovascular function - increased Aβ clearance - decreased blood glucose level - increased ketone concentration	[148]
Rat	Male	5-8 rats per group	93.9% fat, 4.4% protein, and 1.7% carbohydrate	- increased NAD+/NADH ratio in the hippocampus - increased NAD+ levels in the hippocampus - increased nuclear sirtuins activity - increased SIRT1 gene expression in the hippocampus - decreased PARP1 and 8-OHdG levels in the hippocampus	[149]
	Both	1-10 rats per group	75.85% fat, 20.12% protein, 3.85% carbohydrate mixed with MCT oil	- decreased blood glucose level - improved to acquire the correct alternation strategy - improved behaviour on both the elevated figure-8 maze alternation task and a cognitive dual task	[144]
Human		Number of subjects treated	Diet	Functional outcome	Ref*
Trial type	Sex				
Case study	Female	1	Low a carbohydrate/high fat diet, calorie restriction (fasting)	- improved memory with high intensity interval exercise - improved metabolic syndrome biomarkers	[152]
Case study	Male	1	The 10 weeks intervention incorporated a ketogenic diet	- improved memory with high intensity interval exercise - improved metabolic syndrome biomarkers	[151]
Randomized	Female	2	Carbohydrate consumption to less than 50 grams/day	- improved cognition in the patients with HIV	[154]
Randomized, two-phase crossover dietary and exercise trial	Both	12 (8 females and 4 females)	60% fat, 25% protein, and 15% carbohydrate	- improved cognition	[150]
Case study	Female	1	The 10 weeks intervention incorporated a nutrition	- improved memory with high intensity interval exercise - improved metabolic syndrome biomarkers - alleviated the symptoms of insulin resistance and risk induced by mild AD with daily brain training	[155]
Case study	Male	1	A clinically prescribed KD with moderate protein (based on lean mass and activity level) designed to reduce fasting insulin levels	- improved fasting glucose, fasting insulin, and blood lipids in diabetic patient - improved cellular insulin sensitivity in diabetic patient - improved memory, cognition, and verbal fluency in diabetic patient	[153]

* These references were published from 2018.

The mechanism(s) for the beneficial effects of KD are various. For example, KD has similar influence to CR on NAD+ metabolism, AMPK, SIRT1, and antioxidant genes, and activation of PGC-1α which regulate mitochondrial function [144, 156] (Fig. 3). Moreover, beta-hydroxybutyrate (BHB), a ketone body generated by ketogenesis in KD, can rescue mitochondrial function and improve cognitive function [144, 147]. The ketone bodies are known to be used as energy source instead of glucose in KD condition [156]. Furthermore, KD inhibited fatty acid synthesis, glucose metabolism, and protein synthesis, while upregulating peroxisome proliferator-activated receptor α (PPAR α) target gene [96].

The standardization of KD is limited, although KD has been constantly studied in animal models. For example, there was a slight difference in regimen between recent studies [144, 148, 149]. Human studies not only had different dietary regimens, but also consisted mostly of case studies, and therefore were limited to a small sample size [151-153, 155]. Another major limitation has been patient compliance, owing to poor palatability and meagre food choice. Patients were also required to accurately measure all their food portions which introduces subjective bias in the studies [157]. A lack of understanding of potential side effects also exists. Some reported adverse effects of KD include constipation, menstrual irregularities, elevated serum cholesterol and triglycerides, hypoproteinemia, hemolytic anemia, elevated liver enzymes gall-stones, and renal stones [157]. The KD is contraindicated in patients treated with valproic acid which appears to increase the likelihood of adverse events [157]. On the other hand, although there is insufficient data to understand the side effects of KD administration, some effects can be predicted, and the other ones are unusual or caused by long-term treatment [142]. In obese patients, total 83 patients with KD for 24 weeks showed no adverse effects [158]. Moreover, a recent study reported how a low-fat diet or KD can affect motor and non-motor symptoms in PD. Consequently, the KD showed positive effects mostly in non-motor symptoms including cognitive impairment only mild adverse effects [159]. These studies support that KD show beneficial effects without side effects or side effects have a certain predictability.

#### 3.3.3 Promotion of cellular NAD+ anabolism

NAD+ is a critical ‘longevity’ factor which has a major impact on aging hallmarks, including mitochondrial homeostasis, oxidative damage, Ca^2+^ homeostasis, neuronal networks, DNA repair, and inflammation [160]. NAD+ is also associated with sirtuin deacetylases or ‘lifespan extension’ genes involved in transcriptional regulation, the DNA repair protein PARP1, and CD38 which is related to Ca^2+^ homeostasis and immune response [161] but also affects mitochondrial biogenesis through SIRT1/PGC-1α signalling [156]. Thus, NAD+ seems to be a of crucial factor in the aging brain. We were the first to show that NAD+ levels decline with age in most catabolic tissues including the brain. This fact can support the beneficial role of CR and NAD+ in aging. Indeed, CR can increase NAD+ level [162]. as a mechanism to slow down aging. NAD+ levels also decline in neurodegenerative diseases including multiple sclerosis.

NAD+ anabolism in mammalian cells occurs *de novo* from tryptophan (TRYP). NAD+ synthesis through quinolinic acid (QUIN), a kynurenine pathway metabolite, has important immunoregulatory roles [163]. However, overconsumption of TRYP can increase the levels of the putative neurotoxin QUIN which has been associated with the pathogenesis of several neurodegenerative disorders [164]. Therefore, TRYP is an unlikely strategy to elevate NAD+ levels in the clinic.

NAD+ can also be produced via the salvage pathway from NAD+ precursors, nicotinic acid (NA), nicotinamide (NAM), nicotinamide mononucleotide (NMN) and NR [165]. NAD+ can be synthesised from NA via the Preiss-Handler process. However, NA therapy induces some negative adverse effects including significant skin flushing in most individuals below therapeutic doses, thus limiting its widespread clinical use [166]. NAM is generated as a by-product of enzymatic degradation of pyridine nucleotides. While supplementation with NAM raises NAD+ but does not cause flushing, it is not considered an ideal supplement to raise NAD+ due to its enzyme inhibiting (e.g., PARPs, sirtuins, CD38), methyl depleting and hepatotoxic potential [165].

**Figure 4.**
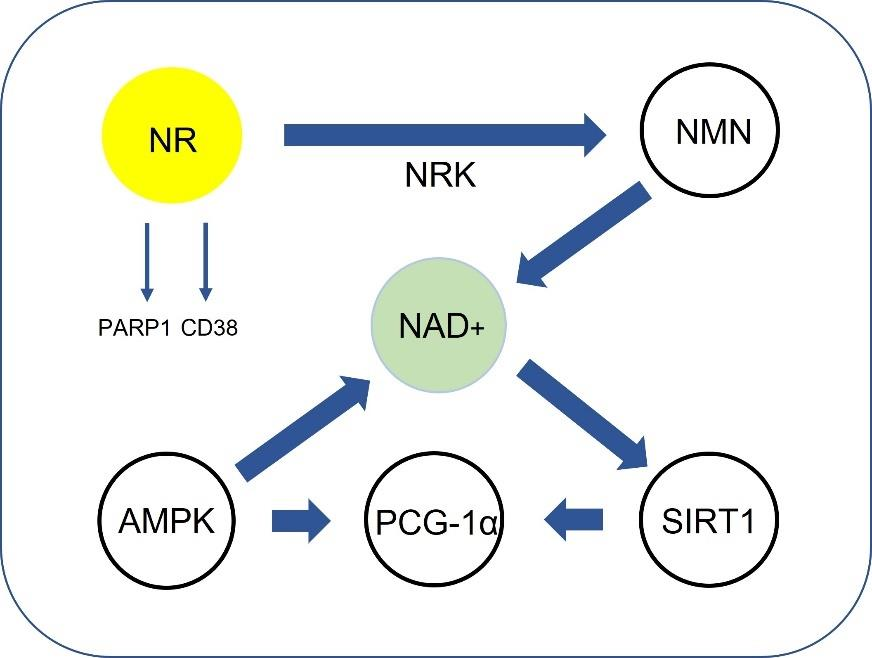
Benefits of NR in brain ageing. The NR pathway is quite unclear. NR is the precursor for NAD+. During the process that converts NR to NAD+, NRK is the important kinase. NAD+ and SIRT1 can activate the PGC-1α. PGC-1α regulates some antioxidant factors. NR is also associated with CD38 and PARP1, which is related to Ca2^+^ transport efflux, DNA damage and has immunogenic roles.

The NAD+ biosynthesis contains NR and nicotinamide mononucleotide (NMN) (Fig. 4). NMN can also be synthesised from NR by the NR kinases, NRK1 and NRK2 [167]. Numerous studies have shown that NMN can attenuate degenerative conditions and slow down age-related cognitive decline [168-173]. For instance, NMN treatment maintained neural stem/progenitor cell population in the aged hippocampus and protected against mitochondrial and cognitive dysfunction in murine models for AD [168-173]. NMN appears to be rapidly absorbed from the gut and into the blood and transported into tissues [174]. The fast pharmacokinetics of NMN has suggested that there is specific NMN transporter that mediates uptake of NMN into the gut and other tissue. Recently, a genetic, pharmacological and kinetic study reported that NMN is dephosphorylated to NR before cellular internalization by the solute carrier family 12 member 8 (Slc12a8) [175]. However, a ‘Matters Arising’ to that article suggested that the analytical methodology and interpretation of those findings were not sound and did not support Slc12a8 as the ‘reclusive’ NMN transporter [176]. As well, NMN may also be neurotoxic and accumulation of NMN may promote axonal degeneration [177].

Naturally, NR mainly exists in avocado, milk, cucumber, and beef [178]. NR is the precursor of nicotinamide adenine dinucleotide (NAD+) [179, 180]. Several studies have continually been undertaken (Table 3). Accordingly, NR increased NAD+ levels, which is related to anti-aging and age-related brain function [180, 181]. NAD+ has beneficial effects on reducing amyloid beta in AD mouse model and neuroprotection in Huntington’s disease (HD) [182]. NR reduced amyloid concentrations induced by high-fat chow diet [183] and lowered the levels of pTau level and amyloid beta plaques in AD mouse models [161, 184]. Through these results, it is assumed that NR might be related to brain aging with NAD+ or other pathways. One of the evidences is that treatment of NR showed recovery of synaptic plasticity and behaviour while NAD+ level and PGC-1α level increased in AD mouse model. NAD+ can regulate PGC-1α via SIRT1, which is associated with AD [180, 185, 186]. Reduction of PGC-1α is related to accumulation of amyloid beta in AD. This means that NR may increase the -expression of PGC-1α through upregulation of NAD+, which may reduce the aggregation of amyloid beta and promote synaptic plasticity in AD models [180, 185, 186]. NR also has influence on recovery of cognitive function in mice having cerebral small vessel disease causing AD [187]. Moreover, NR demonstrated neuroprotective effects in PD mice model [182, 188]. NR is also effective in axonal neurodegeneration in mice. Interestingly, the author identified that NR uses same pathway with NAD+ when preventing the neurodegeneration, but the effect of NR is much higher than that of NAD+ alone [189]. The decline of the dopaminergic (DA) neuron and climbing ability induced by human N370S GBA was mitigated by NR in a fruit fly model (Schondorf et al., 2018). In mice, NR upregulated factors related to aging such as NAD+, SIRT1, and PGC-1α [190, 191], while apoptosis and inflammation were downregulated [161, 190-192]. Furthermore, positive cognitive effects such as learning and memory were observed by NR [161, 183, 184, 192].

**Table 3:** Recent studies regarding the potential benefits of nicotinamide riboside supplementation on brain health.

Animal		Number of animals	Treatment	Dose	Functional outcome	Ref*
Model type	Sex					
Drosophila	-	~ 50 animals per conditions	Oral gavage	500 mM	- alleviated DA neuron loss and the decline in climbing ability in mutant N370S GBA flies which show increased ER stress, an age-dependent loss of DA neuron	[408]
Mouse	Both	5-17 mice per group	Oral gavage	12 mM	- increased the NAD+/NADH ratio in the cerebral cortex - improved learning ability - alleviated decline of memory and working memory in AD mice - decreased pTau levels and pTau/total Tau ration in AD mice	[184]
	Both	10-16 mice per group	Oral gavage	2.5 g/kg	- decreased the number and total area of Aβ plaques in cortex in AD mice - improved selective cognitive impairment in AD mice - decreased chronic brain neuroinflammation in AD mice	[161]
	Male	8 mice per group	Oral gavage	400 mg/kg	- increased the weights of whole brain by 6 weeks - alleviated the increase of amyloid-concentration by high-fat chow diet in the brain. - improved learning and memory	[183]
	Both	3-17 mice per group	Intraperitoneal injection	200 mg/kg	- increased NAD+ levels - decreased apoptosis in the somatosensory cortex and hippocampus - decreased neuroinflammation	[191]
	Male	4-6 mice per group	Oral gavage	100 μg/kg	- increased NAD+ levels in Gulf War Illness mice model - increased SIRT1 levels in Gulf War Illness mice model - increased PGC-1α levels in Gulf War Illness mice model - increased and acetylated PGC-1α levels in Gulf War Illness mice model - decreased brain inflammation in Gulf War Illness mice model	[190]
	Male	5-7 mice per group	Oral gavage	400 mg/kg	- improved alcohol-induced cognition impairment - decreased alcohol-induced inflammatory cytokines in the brain	[192]

* These references were published from 2018.

Manipulation of NAD+ metabolism is promising therapeutic strategy for the management and treatment of age-related cognitive disorders including AD. There is a growing body of evidence to suggest that raising NAD+ levels using NAD+ precursors may reduce some of pathological hallmarks of AD and improve cognitive performance [161, 168, 170-173, 193, 194]. However, apart from NR (which has 9 clinical papers demonstrating safety), safety data for most NAD+ supplements are not available or have not been collected in a systematic manner [178]. NR (as NR Chloride) has been reviewed and authorized by the four leading authoritative regulatory bodies in the world, including the USFDA, Health Canada, the European Food Safety Authority, and the Therapeutic Goods Administration of Australia. Niagen is the only commercially available NR ingredient that has been twice successfully reviewed under FDA’s new dietary ingredient (NDI) notification [195]. A recent randomized, double-blind, placebo-controlled, parallel-arm study examined the safety of chronic NR supplementation and the dosage required to maintain increases in systemic NAD+ levels. In the study, 132 healthy overweight adults were given either placebo, 100 mg, 300 mg, or 1000 mg of Niagen NR daily for eight weeks. The study reported sustained increases (22%, 51%, and 142%) in whole blood NAD+ at 100, 300, and 1000?mg of NR within two weeks and were maintained throughout the duration of the study. No significant differences in adverse events between the NR and placebo-treated groups or between groups at different NR doses were reported [195]. This suggests that NR is orally bioavailable and well tolerated at once-a-day doses of up to 1 gram per day. However, a 51% increase in whole-blood NAD+ was reported within two weeks of commencing supplementation at the recommended dose of 300 mg daily and was maintained for 8 weeks. Further clinical evidence is necessary to confirm the beneficial effects of NR reported in preclinical animal models for neurodegeneration in humans.

**Table 4:** Recent studies on the effect of senolytics in the brain.

Animal		Number of animals	Treatment	Dose	Functional outcome	Ref*
Animal type	Sex					
Mouse	Both	3-5 mice per group	Oral gavage	5 mg/kg dasatinib with 50 mg/kg quercetin	- decreased the number of NFT-containing cortical neurons - decreased gene expression of the NFT-associated senescence genes - improved neurodegeneration in rTg(tauP301L)4510 transgenic mice	[198]
	Both	3-8 mice per group	Oral gavage	50 mg/kg Navitoclax	- attenuated the upregulation of senescence-associated genes in neurodegenerative disease model - attenuated tau phosphorylation in neurodegenerative disease model	[199]
	-	19 mice per group	Oral gavage	50 mg/kg/day PL	- increased the level of NAD+ in vitro assay - attenuated the cytotoxicity of amyloid beta in hippocampal neuron cell - prevented decline of cognition in AD model - attenuated neuroinflammation in the cortex in AD model	[203]
	Female	7-14 mice per group	Oral gavage	50 mg/kg/day PL	- increased adult neurogenesis in the DG of the aged mice	[204]
	Male	5-13 mice per group	Oral gavage	500 ppm (~25 mg/kg/day) fisetin	- prevented the decline of recognition with age in SAMP8 mice - increased behavioural performance and memory in SAMP8 mice	[208]
	Male	5-29 mice per group	Oral gavage	5 mg/kg dasatinib with 50 mg/kg quercetin	- decreased anxiety-behaviour	[409]
	Both	8-16 mice per group	Oral gavage	5 mg/kg dasatinib with 50 mg/kg quercetin	Short term treatment - decreased Aβ-plaque-associated SA-βGal activity in AD model Long term treatment - decreased Aβ-plaque-associated SA-βGal activity in the hippocampus in AD model - decreased Aβ plaque load in the hippocampus in AD model unlike the short-term treatment	[197]
	Male	6 mice per group	Intraperitoneal injection	1.5 mg/kg Navitoclax	- attenuated senescent cells induced by whole brain irradiation - attenuated senescent astrocytes induced by whole brain irradiation - attenuated performance decline induced by whole brain irradiation	[200]
Rat	Male	6 rats per group	Oral gavage	10 mg/kg or 20 mg/kg fisetin	- prevented the behavioural impairment in rotenone induced PD model - increased GSH levels and catalase levels in rotenone induced PD model	[209]

* These references were published from 2018.

#### 3.3.4 Senolytics

Senolytics are a class of small molecules that remove senescent cells, which are affected in age-related diseases. Elimination of senescent cells using senolytics might have anti-aging benefits in the brain. Senolytic agents currently being investigated include piperlongumine (PL), dasatinib, quercetin, fisetin, FOXO4 peptide, ABT-263 (navitoclax), and ABT-737. Some senolytics may be positively related to brain aging and neurodegenerative disease (Table 4) (Baker and Petersen, 2018; Walton and Andersen, 2019). Among them, a senolytic cocktail of dasatinib plus quercetin (D+Q) have been studies in brain aging. In an AD rat model, quercetin had effects on diminishing cognitive deficits [196]. Treatment with D+Q decreased Tau-containing neurofibrillary tangles (NFTs), which is one of the main pathological hallmarks of AD, in a neurodegenerative mouse model as well as senescence-associated β-galactosidase (SA-βGal) which is associated with amyloid beta plaque in AD [197, 198]. Furthermore, D+Q reduced the accumulation of amyloid beta in an AD mouse model [197]. Acute treatment of D+Q diminished senescent oligodendrocyte progenitor cells (OPCs), is related to amyloid beta, with p16 which induce senescent OPCs [197]. The author assumed that decreased aggregation of amyloid beta by D+Q might recover cognitive dysfunction in AD [197]. Treatment with navitoclax also reduced tau phosphorylation in a mouse model which expresses high levels of human tau in neurons [199] and blunted the senescent cell and performance decline induced by whole brain irradiation [200]. PL is known to have anti-inflammatory and anticancer ability [201, 202]. PL administration also increased the level of NAD+ in vitro assay and attenuated amyloid beta in hippocampal neuron[203]. Moreover, treatment of PL improved adult neurogenesis in the DG and prevented or blocked the decline of cognition in AD mouse model [203, 204]. In mice treated with fisetin, improvement of behavioural performance and cognition and reduction in apoptotic neurodegeneration induced by aluminum chloride were observed [205-207]. A recent study illustrated that fisetin oral administration prevented the decline of recognition with age and increased behavioural performance and memory in the senescence-accelerated mouse prone 8 (SAMP8) model of aging and AD [208]. The other group demonstrated that treatment with fisetin attenuated the impairment of behaviour and increased GSH levels and catalase levels in rotenone induced PD model [209] In AD mouse model, Fisetin showed improvement of learning and memory as well as attenuation in tau phosphorylation [210, 211].

**Figure 5.**
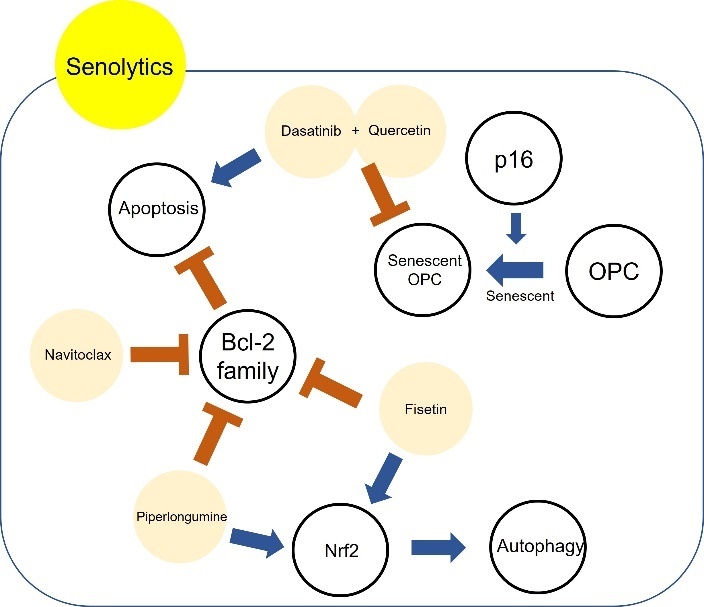
Protective mechanism of senolytics in brain ageing. Piperlongumine, fisetin, dasatinib, quercetin, and navitoclax are included in senolytics. The mechanism of action and effects of senolytics in brain ageing remain obscure. Dasatinib plus Quercetin eliminates senescent OPC. Dasatinib induces apoptosis. Moreover, piperlongumine, fisetin, and navitoclax also induce apoptosis via inhibition of the Bcl-2 family. On the other hand, piperlongumine and fisetin activate autophagy.

The effect of senolytics on senescent cells is dependent on apoptotic pathways such as B-cell lymphoma 2 (Bcl-2) [212] (Fig. 5). PL and Navitoclax are inhibitors of Bcl-2 family proteins which regulate mitochondrial mediated apoptosis, while PL activates autophagy [213, 214]. Fisetin can activate not only the PI3K/Akt/Gsk3β pathway in AD mouse model and Nrf2 but also autophagy [211, 215]. D+Q showed selective elimination of senescent cells in humans and demonstrated little effect on macrophage [216].

While it is anticipated that senolytics are specific to senescent cells, they also have various unwanted side effects since administration is not directed at senescence cells. For example, the release of apoptotic bodies can further stimulate the release of pro-inflammatory proteins that may be cytotoxic to various tissues [217]. More specific side effects such as thrombocytopenia and neutropenia have also been reported following Bcl-2 inhibition [218]. One way to improve the specificity and targeting of senolytics to senescent cells has been through nanocapsulation. These nanocapsules contain enzyme substrates that are overexpressed in senescent cells, allowing the release of senolytics specifically inside senescent cells which then undergo apoptotic cell death. As well, the fate of senescent cells and regenerative processes in the body are nascent in the current literature [219]. Improvement in specificity is important for non-targeted senolytics such as quercetin and fisetin. Moreover, Fisetin higher than effective concentration of senolytic can cause side effects like being cytostatic in proliferating cells [220]. However, the side effects of fisetin are only a little known so far [210]. A recent study illustrated that oral administration of D+Q (D: 100mg/day, Q: 500mg twice daily) for 11 days show no serious side effects [221]. Although these studies can support the safety of senolytic agents, it is not enough to apply to human. The further study what concentration of drugs is effective because the lower concentrations will be ineffective to senolytic and higher concentrations will be toxic.

Although several animal studies have identified beneficial effects and mechanism(s) of action of senolytics, human studies are limited. For example, the effective dosage of senolytics in animal studies may be insufficient to produce desirable effects in humans. While there are several positive findings of using senolytics in animal models, it may be difficult to predict the effects in humans since various interactions can occur in humans. Thus, the studies about adverse effects of senolytics are important.

#### 3.3.5 ‘Young blood’ transfusions

Blood transfusion was considered a means to improve health [222]. The effects of young blood were observed in extending life span and attenuating age-related decline [223, 224]. Blood transfusion was established using two parabiotic parings such as heterochronic parabioses (young and old) and isochronic parabiosis (two young or two old) [225]. Among them, heterochronic parabioses specifically showed positive insights. For example, old mice exposed to young blood showed improvements in learning and memory and showed rejuvenation effects on various tissues including the brain [226, 227], and prevented the decrease in neurogenesis, synaptic plasticity and cognition [228, 229]. Furthermore, young blood plasma transfusion improved memory and cognition in an AD mouse model [230]. Young blood transfusion via heterochronic parabioses are still being studied today (Table 5).

**Table 5:** Recent studies indicating the potential effect of ‘young blood’ transfusion on brain health.

Cell	Serum	Treatment	Functional outcome	Ref*
Cell type				
Human neurons from H1 ES cells	15 days or 12 to 15 months old mice	Cultured in the serum	- increased dendritic branch points of neurons - increased dendritic arbor complexity - increased spine-like outgrowths - increased synapse numbers - increased the functional synaptic connectivity	[231]

Human		Number of subjects infused	Dose	Functional outcome	Ref*
Trial type	Sex				
Randomized, double-blind crossover protocol with 4 once-weekly infusions	Both	9 (Total 18)	1 unit (approximately 250m) plasma from male donors aged 18 to 30 years or 250mL of saline	- adverse events were mild and moderate compare to placebo which means that it is safe, well tolerated, and feasible. - no change in assessments of cognition, mood, functional ability, and default mode network changes	[244]

* These references were published from 2018.

**Figure 6.**
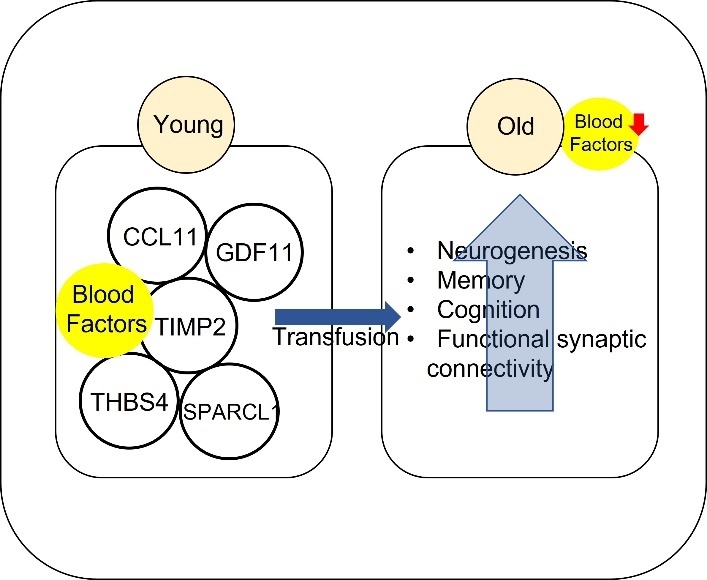
Mode of action of ‘young blood’ transfusion in brain ageing. Blood factors which are decreased with ageing including CCL11, GDF11, TIMP2, THBS4, and SPARCL1. Increasing these blood factors through introduction of young blood in the elderly is aimed at improving neurogenesis, cognition, and synaptic connectivity.

Although the mechanisms behind the success of young blood transfusion are unclear, the beneficial effects may be related to an increase in certain blood-factors such as tissue inhibitor of metalloproteinase 2 (TIMP2), growth differentiation factor 11 (GDF11), C-C motif chemokine 11 (CCL11), thrombospondin-4 (THBS4), and secreted protein acidic and rich in cysteine-like protein 1 (SPARCL1) [227, 231] (Fig. 6). TIMP2 was identified in human umbilical cord plasma [232]. In aged mice, injection of recombinant TIMP2 showed similar effects with injection of umbilical cord plasma to attenuate cognitive decline and TIMP2 knockout mice showed age-related cognitive decline [231, 232]. Secondly, administration of GDF11, which is broadly expressed in the central nervous system (CNS) and detected in human serum [233-235], improved neurogenesis in old mice [236]. Exogenous administered GDF11 (rGDF11) also improved memory and cognition in middle-aged mice [237]. Thirdly, CCL11, which was previously considered to be related to inflammation and immunity [227], increases with age in mice and human [229, 238]. Circulating levels of CCL11 were increased in people with neurodegenerative diseases including AD [239, 240]. Lastly THBS4 and SPARCL1 were enriched in young serum [231]. Both THBS4 and SPARCL1 increase the density of synaptic connectivity, respectively [231].

Since the understanding of young blood components is unclear, there are some limitations. For example, plasma proteins, leucocytes, red cell antigens, plasma and pathogens may cause side effects [241]. This is why plasma infusions are safer for adverse effects than whole blood infusions [225]. For instance, Allergic reactions from mild reactions to anaphylaxis are major cause for concern [242]. Addition side effects can cause the risk of infection and hemolysis in aged with heart failure [243]. In particularly, whole blood infusion can lead to more side effects than plasma infusion [225]. A recent study reported that young donor plasma infusion to patients with AD showed improvement of daily tasks and safety [244]. This study supports what plasma infusion is known to be safe. Moreover, it is difficult to predict how it works, especially in human and limited to small samples. Therefore, the further studies are needed to identify the specific factors and the effects in human. Recently, parabiosis was used to connect young and old mice. The study found that each mouse had equal parts old blood and young blood circulating through it, the young mouse reported negative effects. Old blood drastically decreased hippocampal neuron generation, learning and agility, and liver regeneration in young mice [228]. However, no significant benefits in cognition, agility, or neurogenesis were reported in old mice exposed to young blood. Therefore, improved brain health may not be related to promotion of rejuvenating factors but rather the inhibition of factors in old blood that promote brain aging [245].

#### 3.3.6 Enhancement of adult neurogenesis

Adult neurogenesis (AN) is a process that involves neural stem cell (NSC) maturation, migration, and addition into previously existing neuronal networks in the adult brain [246]. AN is observed in two niches in the CNS including sub-ventricular zone (SVZ) of the lateral ventricles (LVs) and sub-granular zone (SGZ) of the dentate gyrus (DG) [246]. In these neurogenic niches, AN declines with age mainly due to reduction of NSCs and neural progenitor cells (NPCs) [247-249]. AN was also confirmed in the dentate gyrus (DG) of rodents and humans [250] and is associated with brain function including memory and learning [251]. The decline of AN can cause reduction in proliferation and neuronal production, and the reduction might be associated with age-related plasticity and brain repair capacity [252]. Furthermore, AN is associated with impairment of cognition and aging [253, 254]. The decrease in AN is thought to play a vital role in several degenerative diseases such as AD, PD and HD in murine models as well as ‘normal’ aging in rodents and humans [255]. In human hippocampus, NPCs modulate new neurons by stimulating adult hippocampal neurogenesis (AHN) [256]. Moreover, new neurons derived from NPCs have high degree of synaptic plasticity in both SVZ [257]and SGZ [258]. A recent study suggested that mitochondrial dysfunction is associated with a decline in neurogenesis in the SGZ [259].

**Table 6:** Recent studies demonstrating the potential effect of modulation of adult neurogenesis on brain function.

Animal		Number of animals	Treatment	Dose	Functional outcome	Ref*
Animal type	Sex					
Mouse	Male	8 mice per group	Intraperitoneal injection	2 mg/kg IQM316 and melatonin	- promoted AHN by IQM316 and melatonin - induced differentiation of neuronal precursors by IQM316 and melatonin	[260]
	Both	3-12 mice per group	Intraperitoneal injection and hippocampal injection	20 mg/kg P7C3 and 2.0 Pl LV-Wnt3	- promoted AHN by P7C3 and LV-Wnt3 - improved pattern separation memory in male of AD mouse model but not in female of AD mouse model by increasing AHN - no change in other forms of cognition by increasing AHN in AD mouse model - improved pattern separation memory in female of AD mouse model by increasing AHN and BDNF	[261]
	Male	3-6 mice per group	-	-	- decreased p38 expression level with ageing - the reduction of p38 expression is associated with the decline in adult neurogenesis. - prevented the age-related decline in neurogenesis by sustained expression of p38 - regulated neural progenitor cells (NPCs) proliferation in the adult SVZ by p38	[247]
	Male	6-10 mice per group	Mixed with drinking water and then oral gavage	5 mg/kg TCQA	- increased adult neurogenesis in the DG - improved spatial learning and memory in SAMP8 mice	[262]

* These references were published from 2018.

Several recent studies have investigated AN as an anti-aging target (Table 6). Mice injected with 2-(2-(5-methoxy-1H-indol-3-yl)ethyl)-5-methyl-1,3,4-oxadiazole (IQM316), which is a melatonin analog, and melatonin showed upregulation of AHN and differentiation of neuronal precursors [260]. Choi et al (2018) [261] illustrated that the AD mouse model injected with aminopropyl carbazole (P7C3) and lentivirus expressing Wnt3 (LV-Wnt3) to induce neurogenesis promoted neurogenesis and improved pattern separation memory in AD male mice. The p38 MAPKs, which are associated with the decline in AN, prevented age-related decline and regulated NPCs in mice [247]. The 3,4,5-tricaffeoylquinic acid (TCQA), a caffeoylquinic acid derivative, increased AN as well as improved spatial learning and memory in SAMP8 mice, a murine model for accelerated aging [262].

**Figure 7.**
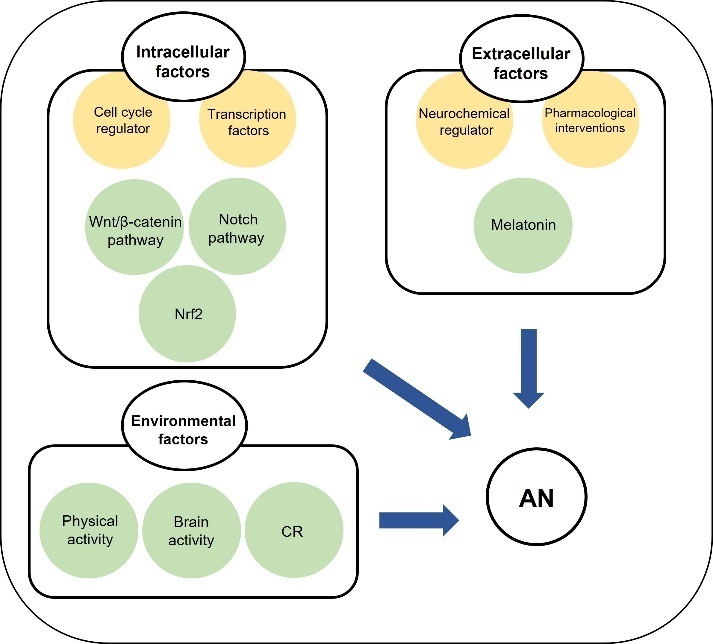
Mechanism of AN in brain aging. AN can be stimulated by intracellular factors, extracellular factors, and environmental factors. These factors can modulate AN.

AN is known to be regulated by intracellular, extracellular and environmental factors [246] (Fig. 7). For example, intracellular factors include cell cycle regulators and transcription factors including Wnt/β-catenin pathway, Nrf2, and Notch pathway, while extracellular factors include neurochemical regulators and pharmacological interventions [246]. Among them, the Wnt/β-catenin pathway is a key regulator [246, 263]. The components related to Wnt/β-catenin pathway are expressed in the hippocampal neurogenic niche [264, 265]. Furthermore, Wnt/β-catenin signaling is considered to be linked to pathological conditions, neurodegenerative diseases, and AN and behavioural decline [246]. As well, N-acetyl-5-methoxytryptam (Melatonin), a neuro-hormone, is known to have neurobiological functions including modulation of AN and mitochondrial function [260]. Melatonin not only regulated proliferation and neurogenesis in the DG of rats [266, 267] but also increased cell survival and dendrite maturation of new neurons in the hippocampus of mice [268, 269].

Although AN is actively studied, the mechanism is broad and unclear. For example, there are various factors to enhance AN, however the understanding of each factor and their connection is insufficient and yet to be confirmed in human clinical trials. Our understanding of neurogenic mechanisms and factors that influence AN has increased significantly in the last decade. For example, we have an extensive understanding on the importance of vasculature and glial cells on AN and how the vasculature-glial-neuronal crosstalk is influenced by several extrinsic factors such as dietary intake and physical activity. Modulation of AN by regulating transcription factors, cytokine release, neurotransmitters and neuropeptide hormones mediated by maintaining a ‘proneurogenic’ lifestyle could delay the onset and reduce the severity of neurodegenerative diseases and promote ‘healthy’ brain aging [270]. Further studies are necessary to evaluate the clinical utility of well-defined approaches in aging and neurodegenerative conditions.

#### 3.3.7 Stem cell therapy

**Figure 8.**
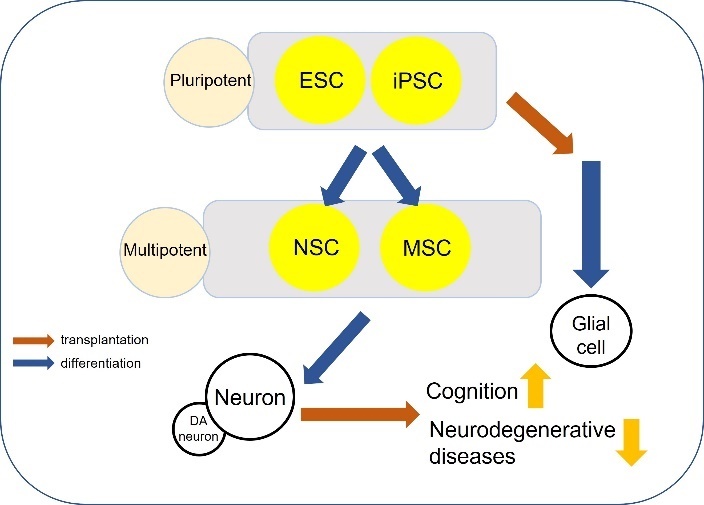
Potential role of stem cell therapy in brain ageing. ESC, iPSC can differentiate into various cell types including NSC and MSC since they are pluripotent. Although MSC is mainly differentiated into osteoblasts, chondrocytes, myocytes, and adipocyte, MSC can also differentiate into neurons. Transplantation of neurons derived from NSC or MSC can improve cognition and attenuate degenerative diseases. ESC and iPSC can be directly transplanted for treatment then differentiate into other brain cells such as glial cell.

Cell therapies have emerged as potential treatments for neurological disorders and aging [271-296]. Stem cells can proliferate and differentiate into multiple cellular lineages. There are different classifications of stem cells, which are used therapeutically, including embryonic stem cells (ESCs), induced pluripotent stem cells (iPSCs), NSCs, and mesenchymal stem cells (MSCs) [297] (Fig. 8). ESCs, which is pluripotent and self-renewal, can generate neural cells such as neurons, oligodendrocytes and glial cells [298], and has been suggested as a potential therapeutic stem cell [299]. Transplantation of ESCs showed improvement in cognition in rodent models of brain injury [300] and improved behavioural performance in a PD primate model using ESCs derived from both primates and humans [301, 302]. Moreover, transplantation of ESC-derived basal forebrain cholinergic neurons (BFCNs) improved learning and memory in AD mouse model [303].

iPSCs are also potential therapeutic stem cells because of their self-renewal capacity and their ability to differentiate using a 3-phase reprogramming technique including initiation, maturation and stabilization [304, 305]. Transplantation of iPSCs derived from mouse skin fibroblasts induced differentiation into glial cells and improved cognition and decreased plaque depositions in an AD mouse model [306]. iPSC therapy improved behavioural performance in a PD rat model [307] and in PD monkey model [308]. Furthermore, iPSC-derived DA neurons were suggested to be suitable for transplantation because their characteristic is similar to human DA midbrain neurons [309]. NSCs, which can be derived from both ESCs and iPSCs, have the ability of self-renewal and multipotent potential [310]. In AD rodent, NSCs transplantation showed decline in neuro-inflammation, tau and Aβ pathology [311, 312] and improvement in AN, synaptogenesis, and cognition [312-314]. Transplantation of NSCs also improved motor impairment in HD animal models [315].

MSCs not only differentiate into various cells including cartilage cells, muscle cells, fat cells, bone cells, and connective tissue cells but also have low immunogenicity [316]. It is generally accepted that MSCs do not exert their beneficial actions through direct differentiation into neural tissue, but rather by acting as trophic mediators releasing immune modulatory, proangiogenic, and/or pro-neurogenic factors [317]. Additional mechanisms involved in paracrine signalling promoted by MSCs include the secretion of specific cytokines [318] and the transfer of extracellular vehicles (EVs) or even of healthy mitochondria to cells with impaired mitochondrial function [319-321].Recent advances in biomedicine have led to a growing interest in using stem cells as cellular vectors for disease modelling, drug discovery, drug toxicity, and regenerative medicine. Importantly, MSCs have a greater proliferation capacity in vitro with no time limit [322]. They also have immunomodulatory properties, and it was reported recently that they are capable of impairing NK-cells’ function to prevent graft rejection. Transplantation of MSCs reduced tau phosphorylation and improved AN and cognition in vivo [297]. Human MSCs not only attenuated accumulation of amyloid beta but also improved synaptic transmission and memory in an AD mouse model [323, 324]. Moreover, MSCs transplanted showed improvement in locomotion and cognition in aged mice and prevented the accumulation of amyloid beta as well as improved learning and memory in an AD rodent model [325]. Recently, some studies using stem cell therapy in various animal model reported (Table 7). In mice, human NSCs showed improvement of behavioural performance in HD model [326]. Human MSCs improve spatial memory in aged rats [327] and functional neurological recovery in a swine model of traumatic brain injury and hemorrhagic shock [328]. human pathogenetic embryonic stem cells (hPESCs), which is generated from activated oocytes without sperm fertilization, can differentiate DA neuron, and improve locomotive performance in a PD primate model [329].

**Table 7:** Recent studies utilising stem cell therapy on brain function.

Animal		Number of animals	Cell type	Functional outcome	Ref*
Animal type	Sex				
Mouse	Both	5-8 mice per group	hNSCs	- Improve behavioural performance such as rotarod, pole test, and grip strength in HD model - reduced hyperexcitable input from cortex to striatum after addition of the GABA_A_ receptor antagonist in HD model - increased BDNF levels in HD model.	[326]
Rat	Female	8 rats per group	hMSCs	- improved spatial memory accuracy in aged rats - increased neuroblasts in aged rats - decreased the number of reactive microglial cells in aged rats - restored presynaptic protein level in aged rats	[327]
Pig	Female	5 pigs per group	hMSCs	- hMSCs derived exosome attenuate neuronal injury in traumatic brain injury and haemorrhagic shock model. - hMSCs derived exosome improve functional neurological recovery in traumatic brain injury and hemorrhagic shock model.	[328]
Monkey	Male	10	hPESCs	- the transplantation of hPESCs derived DA neuron was relatively safe without tumor in PD model. - the transplantation of hPESCs derived DA neuron improved locomotive performance in PD model.	[329]

* These references were published from 2018.

Using neurons derived from the patient’s stem cell can relatively be safe from immunorejection. It was reported recently that MSCs are capable of impairing NK-cells’ function to prevent graft rejection. Despite their long-term survival after transplantation, the MSC are nontumorigenic and are safe and effective for cell-based therapy [322]. However, further investigations are needed to alleviate differentiation of tumors or teratomas. Although stem cells derived from various sources have shown positive effects in cognition and neurodegenerative diseases in animal model, studies in humans are limited, and clinical trials are warranted.

It has been established that the main tenet for MSCs to exert a dynamic homeostatic response that supports tissue preservation and function recovery is the generation of exosomes [330]. The main mechanism by which MSCs mediate this activity is not through cellular implant and its subsequent differentiation, but the paracrine activity of the secretome [331]. This phenomenon was demonstrated in studies where conditioned medium of MSCs was administered and therapeutic effects similar to those already reported for MSCs were produced in different animal models of diseases [330]. Exosomes encapsulate and transfer several functional molecules including proteins, lipids and regulatory RNA which can modify cell metabolism. More than 730 proteins have been identified in MSC-derived exosome, including specific cell type markers and others that are involved in the regulation of binding and fusion of exosomes with adjacent cells. Additionally, factors that promote the recruitment, proliferation, and differentiation of other cells such as neural stem cells have also been identified. As well, several miRNAs have been found in exosomes, which regulate neural remodeling and angiogenic and neurogenic processes [332]. Therefore, the use of exosomes could be part of a strategy to attenuate irregular pathology and cognitive deficits and promote neural replacement and plasticity in AD with limited adverse effects and immunorejection.

The safety of stem cell therapy is unclear because unwanted and uncontrolled differentiation could be observed. For example, stem cell could differentiate to undesired tissue after transplantation then it may cause tumors [333]. Nevertheless, stem cell therapy still has possibilities to develop as an application for diseases. The adverse effects mentioned above such as development of teratoma could be prevented by screen them for the presence of undifferentiated cells before injection. For example, a study reported that they did not observed teratomas in over 200 animals using the procedure [334]. Furthermore, the other group study showed that serious side effects including adverse proliferation, tumorigenicity, and ectopic tissue formation were not observed after transplantation of ESC-derived retinal pigment epithelial [335]. These studies can support that stem cell therapy can be available with safe under controlled condition. Several studies confirmed the safety of MSC therapy[336-340]. And MSC also showed safety in patients with TBI and neurodegenerative diseases including multi sclerosis and ischemic stroke patients [341-343]. However, it is insufficient data for clinical application. As a result, it will be necessary to further study about safety and efficacy of stem cell therapy.

**Table 8:** Recent studies about association between vascular factors and brain health.

Animal		Number of animals	Treatments	Functional outcome	Ref*
Animal type	Sex				
Mouse	Male	3-8 mice per group	-	- Increased levels of Aβ in the hippocampus of hypertension mouse model - Increased levels of phosphorylated tau protein in the hippocampus of hypertension mouse model - damaged hippocampus related to learning and memory in hypertension mouse model	[362]
Pig	Male	3-27 pigs per group	-	- Increased levels of Aβ in the hippocampus of hypertension pig model which was induced by abdominal aortic constriction - Increased levels of phosphorylated tau protein in the hippocampus of hypertension porcine model	
Human		Number of subjects	Treatments	Functional outcome	
Trial type	Sex				
-	Both	2,004	Antihypertensive Hypoglycemic agents	- showed positive association between antihypertensives and cognition in older adults with hypertension - showed positive association between cognition and dose of antihypertensives and hypoglycemic agents in older adults with both hypertension and diabetes mellitus type II	[364]
-	Both	3201	-	- observed poor global cognitive and memory performances in hypertensive patients compared to non-hypertensive patients	[363]
Retrospective cohort	Both	116	Antihypertensive	- decreased the risks of AD and dementia by combination of statin and an antihypertensive	[365]
Multi-centre cluster randomized controlled	Both	412	Antihypertensive	- improved cognition in people with dementia	[366]

* These references were published from 2018.

#### 3.3.8 Vascular risk reduction

Numerous studies have shown the early role of vascular factors during the prodromal stage of cognitive impairment, parkinsonism. The Vascular hypothesis assumes that vascular risk factors are one of the major considerations for brain aging and neurodegenerative diseases. Many studies have reported that vascular risk factors such as hypertension, hypercholesterolemia, obesity, and diabetes are associated with cognitive dysfunction and dementia including AD [344-346]. For instance, vascular risk factors in mid-life, but not late-life, showed interaction with amyloid deposition [347]. Furthermore, people with diabetes mellitus showed an increase in the risk of cognitive impairment and dementia in the elderly [348]. In particular, the control of blood pressure, which is an important and essential factor for homeostatic control of the living organism, can impact the aging brain such as cognitive impairment [349, 350].

Hypertension has been associated with cognitive impairment or neurodegenerative diseases. Numerous studies reported that hypertension is related to cognitive decline, mild cognitive impairment, dementia, and neurodegenerative diseases such as AD and PD [351-355]. In animal studies, hypertension affected the deposition of brain amyloid, thereby supporting the association between hypertension and brain amyloid [347]. Hypertension is also related to a significant increase in excess ROS [356, 357]. Overall, hypertension may represent a ‘hidden brain risk’ and brain health can be improved by lowering blood pressure.

**Figure 9.**
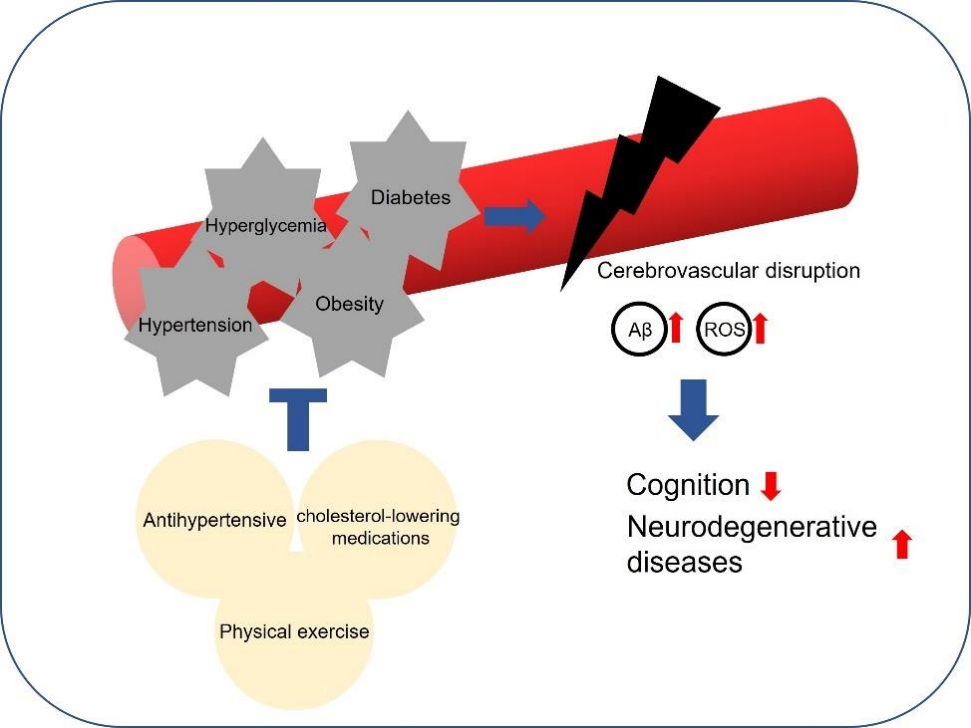
Association between vascular risk factors and cognition as well as neurodegenerative diseases. Vascular risk factors such as hypertension, hyperglycaemia, diabetes, and obesity can increase Aβ and ROS via cerebrovascular disruption. Antihypertensives and cholesterol-lowering medication might improve cognition and prevent neurodegenerative diseases through their effects on vascular risk reduction.

Nevertheless, the molecular mechanism(s) of how these vascular risk factors impact brain health or neurodegenerative diseases is still unclear. However, there have been some concerted efforts to understand how the vascular risk factors can affect the brain and neurodegenerative diseases (Fig. 9). For instance, cerebrovascular disruption such as blood brain barrier breakdown and resting cerebral blood flow reduction, induced by vascular risk factors, can lead to the accumulation of neurotoxic molecules in the brain [358]. In particular, breakdown of the blood brain barrier, which controls the entry of blood-derived products and pathogens into the brain, is observed in mild cognitive impairment and AD [359, 360]. Moreover, vascular dysfunction can lead to increases in Aβ levels and ROS [356, 357, 361]. A recent study demonstrated increases in the levels of Aβ and damage to the hippocampus associated with learning and memory impairments in a hypertension mouse model. As well, increased levels of Aβ and phosphorylated tau protein were reported in the hippocampus of a hypertension porcine model which is induced by abdominal aortic constriction [362]. In humans, hypertensive patients showed poor global cognitive and memory performance compared to non-hypertensive participants [363]. A strong correlation was observed between cognitive impairment in older adults and incidence of hypertension [364].

**Table 9:** Recent studies of non-pharmacological strategies on brain function.

Animal		Number of animals	Intervention	Functional outcome	Ref*
Animal type	Sex				
Mouse	Male	12 mice per group	Treadmill	- decreased Aβ deposition in the hippocampus of AD model - decreased Aβ levels in the hippocampus of AD model - increased autophagy in the hippocampus of AD model	[399]
	-	12 mice per group	Treadmill	- improved learning and memory in AD model - improved mitochondrial fission and fusion balance in AD model - decreased oxidative stress and Aβ levels in the hippocampus of AD model	[400]
	Male	12 mice per group	Treadmill	- attenuated Aβ deposition in the hippocampus of AD model - decreased Aβ levels in the hippocampus of AD model - decreased Aβ production in the hippocampus of AD model	[398]
	Male	25 mice per group	Swimming	- decreased Aβ an P-Tau in the cortex and hippocampus of AD model - attenuated cognitive impairments in AD model	[403]
	Female	10 mice per group	Treadmill	- decreased soluble Aβ levels in the hippocampus of AD model	[397]
	Both	8-10 mice per group	Running wheels	- improved performance in rotarod of AD model - decreased Aβ plaque load in the hippocampus of AD model	[402]
Rat	Female	6-8 rats per group	Running wheels Swimming	- improved locomotion impairment in AD model - improved learning ability in AD model - decreased Aβ level in AD model - prevented the decrease of neurotrophic factors such as NGF and BDNF in AD model - ameliorate oxidative stress in AD model	[401]
	Male	20 rats per group	Environmental enrichment Social enrichment Anaerobic exercise	- prevented impairments in object recognition memory by environmental enrichment and anaerobic exercise - prevented impairments in social recognition memory by each intervention - increased total antioxidant capacity by social enrichment	[404]
	Male	14 rats per group	Treadmill	- prevented Aβ-induced impairment of spatial learning and memory in MWM test - decreased Aβ load and soluble Aβ levels in the hippocampus and plasma of AD model - increased Aβ-degrading enzymes levels in the hippocampus of AD model	[396]
Human		Number of subjects	Intervention	Functional outcome	Ref*
Trial type	Sex				
Prospective, randomized controlled	Both	13	Dance Therapy	- showed potential to improve motor symptoms in PD patients	[384]
Randomized controlled	Both	288	Music Therapy	- improved verbal fluency test score in mild AD patients - improved psychiatric and behavioural symptoms in severe AD patients	[383]

* These references were published from 2018.

One hot topic in preventing neuro-degenerative diseases such as dementia is optimizing lifestyle and vascular risk factors during middle age, some of which through therapeutic strategies. For instance, pharmacological control of hypertension in middle aged or younger old adults lowered the incidence of dementia in one study [353]. Another study reported reduction in the risks of AD and dementia when antihypertensives were used in combination with statins [365]. Moreover, the treatment of antihypertensive showed improvement in cognition patients with dementia [366]. Recently, the effects of combination of antihypertensive and hypoglycemic agents against cognitive decline has been demonstrated. The study showed a positive correlation with cognition in older patients with both hypertension and diabetes mellitus type II [364].

As well, high levels of cholesterol in plasma is related to risk of AD [367]. *In vivo* and in vitro studies have demonstrated that high levels of cholesterol in blood may increase the production and deposition of Aβ in AD [368, 369]. Treatment with statins, which are cholesterol-lowering medications, has been reported to reduce the production of Aβ in high cholesterol-fed animal models [370]. Taken together, these results indicate that vascular risk reduction may be an effective therapeutic strategy for the promotion of brain health.

Metformin is a medication originally used for type 2 diabetes. Recent studies have also found that metformin has a positive effect on cardio- vascular protection [8,9,10,11,12]. Metformin also lowers risk factors for cardiovascular disease such as blood fats [13,14,15], body weight and blood pressure. Some experiments have highlighted the possibility of using metformin for anti-aging [72, 73]. Metformin has similar effects to CR regulating pathway and genes related to aging [72-75], However, recent studies reported side effects and sex-dependent variability of metformin [76-78]. Metformin had no effect on female mice with neuropathic pain, whereas it inhibited microglial activation in male mice [78]. Neurogenesis of neuronal precursor cells, an essential factor for repairing brain injury, is improved only in female mice [76]. In humans, diabetic patients with metformin had a higher risk of developing PD and AD than non-metformin treated individuals [79, 80]. On the other hand, metformin has shown positive effects on AD and PD in animal models [74, 81-83]. These results are paradoxical compared to the results in *C. elegans* and mice.

#### 3.3.9 Non-pharmacological therapies

Non-pharmacological interventions have also been studied to prevent brain aging and neurodegenerative diseases. There are various non-pharmacological interventions such as physical activity and cognitive stimulation. Music therapy is one of the multi-domain cognitive stimulation approaches, which has shown improvement in memory and reduction of negative cognitive symptoms in AD [371]. On the other hand, physical activity is especially considered a common and affordable way to improve neurological health [372]. It has been reported that physical activity can improve cognitive function such as learning and memory and prevent neurological damage in animals and humans [373-376]. Moreover, physical exercise has been proposed as a therapeutic strategy to attenuate age-related neurodegenerative diseases [377, 378]. Physical activity is universal prescription for AD or PD to improve cognitive function and mental health as well as reduce the risk of other degenerative diseases [379, 380]. Among these physical activities, dance therapy and aerobic exercise have shown positive effects. For example, dance therapy, which involves the use of sound and motor movement for cognitive stimulation, had preventive ability in cognitive degradation [371, 381, 382]. In humans, dance therapy showed its potential to use as an intervention for improvement of motor symptoms in PD patients. Furthermore, music therapy improved verbal fluency in mild AD patients as well as psychiatric and behavioural symptoms in patients with severe AD [383, 384]. Aerobic exercise can not only generate structural changes in the brain but also improve cognition [385, 386].

**Figure 10.**
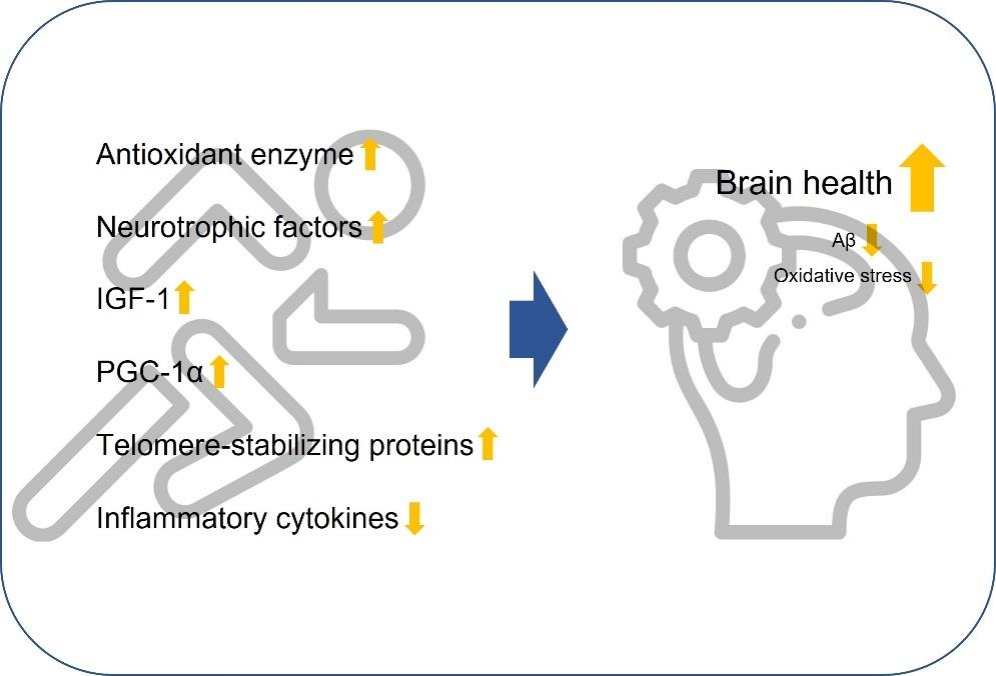
Positive effects of non-pharmacological strategies in brain health. Among non-pharmacological strategies, physical activity can not only increase antioxidant enzymes, neurotrophic factors, IGF-1, PGC-1α, and telomere-stabilizing proteins but also reduce inflammatory cytokines, which might improve brain health.

Although the mechanisms to explain how physical activity improves cognition and reduce neurodegenerative disease risk are still unclear, there are many hypotheses (Fig. 10; Table 9). For example, physical activity can affect ROS and redox balance through upregulation of antioxidant and oxidative damage repair enzymes, consequently leading to a reduction in oxidative stress geeration and improvements in brain function [387-389]. In animal and human studies, physical activity also increased the levels and function of several neurotrophic factors such as BDNF, and nerve growth factor (NGF) as well as the expression of IGF-1 and PGC-1α [376, 390]. Furthermore, physical activity has been shown to increase the levels of telomere-stabilizing proteins and reduce the expression of inflammatory cytokines such as IL-1β or TNF-α [391-393]. Overall, physical activity can not only improve redox status increasing neurotrophic factors and enzymes related to antioxidant, but also protect against cellular senescence through telomere-stabilizing proteins. With respect to its effects in AD, physical activity showed enhancement of Aβ degrading enzymes, thereby reducing Aβ plaques [394, 395].

Other forms of physical activity have demonstrated positive effects on brain aging and neurodegenerative diseases. For instance, exercise using treadmill showed a significant decline in oxidative stress and Aβ levels in the hippocampus of an AD rodent model [396-400]. Moreover, physical activity also increased autophagy and improved learning and memory in an AD mouse model [399, 400]. Additionally, swimming and running wheels were reported to not only decrease Aβ levels but also improve cognition and motor activity in an AD rodent model [401-403]. Apart from the physical activity, environmental and social enrichment additionally showed improvement in recognition memory in rats [404]. These findings provide insight into the potential mechanisms of physical activity and other non-pharmacological therapies against brain aging and neurodegenerative diseases.

### 3.4. Anti-aging limitations

The development of anti-aging drugs is an enormous challenge but of great desire to humans. However, there are several limitations to developing anti-aging drugs. One of the challenges is that it takes a considerably long-time to prove aging processes are attenuated in humans and would require several generations of researchers with immense funding working on the project in a large multi-sited population [67]. Moreover, plenty of clinical trials are essential to identify whether data from preclinical animal models can be translated to humans. Traditionally, in vivo studies have been performed using animals with phenotypically accelerated aging or prolonged longevity, transgenic, mutant, and knockout models that focus on a single gene’s role, to generate reproducible results [68, 69]. Due to their short lifespan, inbred laboratory rodents, particularly rats and mice (e.g. senescence accelerated mice - SAM), are used as models to investigate the effects of intrinsic and extrinsic factors on lifespan [69]. However, this is quite limited since these inbred models do not provide significant genetic diversity to be comparable to humans and correlate poorly with human conditions. To date, more than 150 clinical trial candidates to attenuate inflammation in critically ill patients have failed due to over-reliance on inadequate animal models [70]. Another problem is that humans consume various types of food and have different lifestyles, so it is hard to recognize if anti-aging ingredients have synergistic or adverse effects [71]. The aging phenotype is also affected by individual variability i.e., sex, tissues function and socio-economic status [71, 72].

## 4. Conclusion

As the aging population continues to expand, slowing and/or reversing the impact of aging on the brain is becoming important to maintain a good quality life. There are several anti-aging strategies currently under investigation. Some of these strategies have shown considerable promise for improving brain aging. For example, CR is actively studied as an effective intervention, and many studies contribute to understanding the mechanism of CR on the brain. Although the mechanism(s) are still controversial, there are several hypotheses to explain how CR can reduce age-related decline. Moreover, several mechanisms of CR overlap with other strategies including the ketogenic diet and raising NAD+ levels. Pre-clinical studies have shown that elimination of senescent cells using senolytics, transfusion of plasma from young blood, promotion of cellular NAD+ levels using NAD+ precursors such as NR, neurogenesis and BDNF enhancement through specific drugs are promising approaches to prevent age-related neurocognitive disorders. However, these approaches will require critical assessment in clinical trials to determine their long-term efficacy and lack of adverse effects on the function of various tissues and organs. For example, studies exploring KD are largely limited to case studies [151-153, 155]. On the other hand, approaches such as NR treatment are ready for large-scale clinical trials, as it is non-invasive, safe, and orally bioavailable. Stem cell therapy, such as MSCs were able to improve brain function in the aged or AD brain. However, further advances in stem cell therapy are required for promoting successful brain aging. Clinical relevance of stem cells is dependent on further clinical trials to examine the safety and efficacy of stem cell products.

Several dietary interventions have shown promise. A potential naturally occurring phytochemical is resveratrol (RV) [72]. RV is one of the most well-known polyphenols demonstrating anti-aging affects through Caloric restriction (CR) and other mechanisms [84]. In the brain, RV reduces oxidative stress which is a vital factor for brain aging [85]. Moreover, RV has neuroprotective effects in PD rats [86, 87], accelerates recovery of peripheral infection in rats [87, 88], and reduces amyloid beta accumulation in AD patients [89-91]. Although the potential effects of RV for preventing brain aging have been well demonstrated in humans, there are some limitations in humans. It is safe to take up to 5 g a day, however Intake of overly high doses of RV may cause some minor side effects [92]. In addition, the effects of RV are diverse in the human body, but the drug has low bioavailability, whereas designed drugs influence a single target with high affinity. Another challenge is that absorption rate of pure RV is quite lower than naturally occurring RV [67].

As well, NAD+ precursors, including NAM, NA, TRYP, NR and NMN can be obtained from food. NA and NAM are found in eggs, fish, a variety of meats, dairy products, certain vegetables, and whole grains. NR is abundant in milk. Foods that contain NMN include broccoli (0-25-1.12 mg/100 g), avocado (0.36-1.60 mg/100 g), and beef (0.06-0.42 mg/100g) [405]. Ingested NAD+ may be broken down to a number of NAD+ precursors including NR and NMN via the catalytic activity of enzymes in the intestines and/or gut bacterial nicotinamidases [406]. NAD+ precursors can be absorbed from a variety of foods in the diet.

A greater understanding and refinement of pharmacological and non-pharmacological anti-aging strategies to meet individual needs are essential to maintain optimal brain function and extend the health span to promote a global ‘healthy’ aging population.
